# Expression of the FGFR2 mesenchymal splicing variant in epithelial cells drives epithelial-mesenchymal transition

**DOI:** 10.18632/oncotarget.6706

**Published:** 2015-12-21

**Authors:** Danilo Ranieri, Benedetta Rosato, Monica Nanni, Alessandra Magenta, Francesca Belleudi, Maria Rosaria Torrisi

**Affiliations:** ^1^ Istituto Pasteur-Fondazione Cenci Bolognetti, Dipartimento di Medicina Clinica e Molecolare, Sapienza Università di Roma, Rome, Italy; ^2^ Azienda Ospedaliera S. Andrea, Rome, Italy

**Keywords:** FGFR2, epithelial-mesenchymal transition, human keratinocytes

## Abstract

The FGFRs are receptor tyrosine kinases expressed by tissue-specific alternative splicing in epithelial IIIb or mesenchymal IIIc isoforms. Deregulation of FGF/FGFR signaling unbalances the epithelial-stromal homeostasis and may lead to cancer development. In the epithelial-context, while FGFR2b/KGFR acts as tumor suppressor, FGFR2c appears to play an oncogenic role. Based on our recent observation that the switching of FGFR2b versus FGFR2c induces EMT, here we investigated the biological outcome of the ectopic expression of FGFR2c in normal human keratinocytes. Morphological analysis showed that, differently from FGFR2b overexpression, the forced expression and activation of FGFR2c drive the epithelial cells to acquire a mesenchymal-like shape and actin reorganization. Moreover, the appearance of invasiveness and anchorage-independent growth ability in FGFR2c transfected keratinocytes was consistent with the potential tumorigenic role proposed for this receptor variant. Biochemical and molecular approaches revealed that the observed phenotypic changes were accompanied by modulation of EMT biomarkers and indicated the involvement of EMT transcription factors and miRs. Finally, the analysis of the expression pattern of discriminating markers strongly suggested that activation of FGFR2c triggers a process corresponding to the initiation of the pathological type III EMT, but not to the more physiological type II EMT occurring during FGFR2b-mediated wound healing.

## INTRODUCTION

The fibroblast growth factor receptor (FGFR) family includes four highly conserved transmembrane receptor tyrosine kinases (FGFR1-4) controlling many physiological key processes such as cell proliferation, differentiation, migration and survival [1, 2]. The FGFR structure consists of an extracellular domain containing three immunoglobulin-like (Ig) loops (IgI-III), a transmembrane helix and two intracellular tyrosine kinase (TK) domains. The alternative splicing of the IgIII loop in FGFR1-3, generates the FGFRIIIb or the FGFRIIIc isoforms, which are mainly expressed in epithelial and mesenchymal tissues respectively and which determine the ligand specificity [1]. Since also the FGF expression patterns are tissue-specific, FGF/FGFR signaling appears to be a finely controlled and context-dependent paracrine mechanism whose deregulation unbalances epithelial-stromal homeostasis and might drive cancer development and progression.

Although the deregulation of FGF/FGFR axis is known to play oncogenic roles in some tissue contexts, in others the FGFR activation reveals an opposite tumor suppressive outcome [3, 4]: this is the case of FGFR2, whose epithelial isoform FGFR2b/KGFR, controlled by the epithelial splicing regulatory proteins 1 and 2 (ESRP1 and ESRP2) [5] is down-regulated in several carcinomas [1, 3] and it has been proposed to exert a tumor suppressive role *in vitro* and *in vivo* [6, 7]. In agreement with this postulated role, previous studies from our group have demonstrated that FGFR2b expression and signaling induce differentiation in keratinocytes and that the receptor forced overexpression increases, while its silencing reduces, this process [8, 9]. In the other hand, the out-of-context expression of the mesenchymal variant FGFR2c has been found in many different epithelial tumors [1, 3, 4]. Although several studies have suggested for the FGFR2c isoform a possible important role in the early steps of carcinogenesis [10–14], other reports indicated for this receptor isoform a role in the latter stages of tumor progression and in the metastatic cascade [15]. In light of these not conclusive evidences, the specific impact of FGFR2c expression, particularly in non-tumorigenic epithelial cells, remains to be clarified.

Interestingly, altered FGFR isoform switching is involved in epithelial-mesenchymal transition (EMT) and cancer progression [13, 16, 17] and a very recent study from our group has shown that the switch from FGFR2b to FGFR2c could be the key event that triggers EMT in normal human keratinocytes [18].

Based on the different features and biomarkers involved, EMT has been classified into three distinct processes [19, 20]: the EMT type III (commonly associated with cancer progression and simply named EMT) is characterized by the migration of isolated mesenchymal-like cells and is clearly distinct from the EMT involved either in embryogenesis (type I) and in adult tissue regeneration (type II), both accompanied by a collective cell movement in which cell-junctions (and consequently E-cadherin expression) are conserved [21, 22]. Although the migratory effect of FGFR2b expression and signaling during re-epithelialization is well-established [23, 24], the biological outcome and the possible mode of cell movement induced by the FGFR2c ectopic expression and activation in human keratinocytes remain to be investigated. Taking advantage from the use of a human keratinocyte cell line HaCaT stably or transiently expressing the two FGFR2 isoforms, we found that, differently from FGFR2b overexpression, the ectopic expression of FGFR2c induces morphological and cytoskeletal changes, a gene reprogramming and an invasive behavior that are reminiscent of type III EMT.

## RESULTS

### The ectopic expression of FGFR2c changes the cell morphology and behaviour

**Figure 1:**
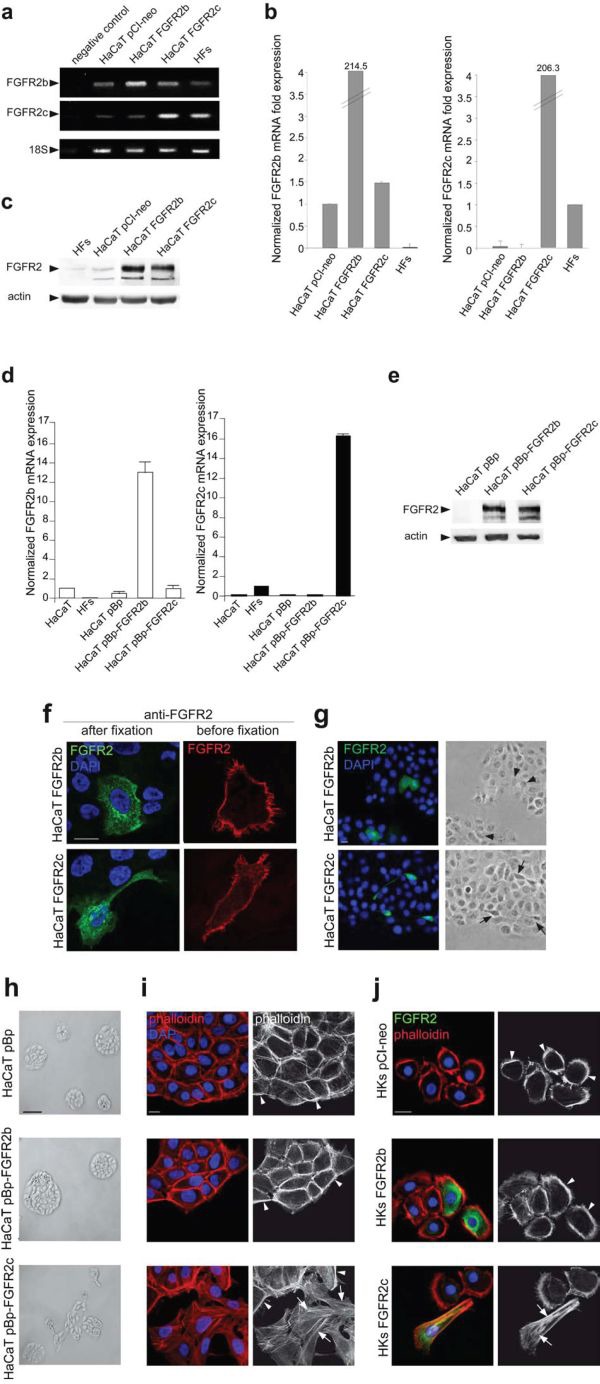
The ectopic expression of FGFR2c affects keratinocyte morphology and their growth mode HaCaT cells were transiently transfected with pCI-neo expression vectors containing FGFR2b or FGFR2c variants (HaCaT FGFR2b and HaCaT FGFR2c) or transduced with pBp retroviral vectors containing the two FGFR2 isoforms (HaCaT pBp-FGFR2b and HaCaT pBp-FGFR2c respectively). HaCaT, HaCaT pCI-neo and HaCaT pBp cells or a primary culture of human dermal fibroblasts (HFs) were used as controls. (**a, b, d**) Qualitative PCR (**a**) and quantitative real-time RT-PCR (**b**, **d**) show that FGFR2c mRNA expression is evident only in HaCaT cells ectopically expressing FGFR2c or in HFs; FGFR2b mRNA expression is detectable at different levels in all HaCaT cells, but not in HFs. 18S rRNA was used as internal control. (**c**, **e**) Western blot analysis, performed using anti-Bek antibodies, shows that the 140 KDa band corresponding to the molecular weight of FGFR2 is more evident in cells transfected or transduced with one of the two FGFR2 variants. The equal loading was assessed using anti-b actin antibody. (**f**) Immunofluorescence analysis was performed in cells incubated at 4°C with anti-Bek antibodies before cell fixation, to selectively stain plasma membrane receptors, or after fixation and permeabilization, to simultaneously visualize the FGFRs intracellularly on the cell surface. Nuclei were stained with DAPI. Receptor signals are localized on the cell plasma membranes and in intracellular dots; only the cells expressing the FGFR2c isoform appear elongated. Bar: 10μm (**g**) Immunofluorescence coupled to phase contrast analysis shows that FGFR2b expressing cells appear polygonal and packed (arrowheads), while FGFR2c expressing cells are spindle-shaped and detached from the neighboring ones (arrows). Bar: 10μm (**h, i**) Low magnification phase contrast microscopy (**h**) coupled to fluorescence analysis using TRITC-conjugated phalloidin (**i**) was performed in stably transduced cells. HaCaT pBp and HaCaT pBp-FGFR2b cultures grow in packed colonies (**h**), display a cobblestone shape and actin cytoskeleton organized in cortical bundles (**i**, arrowheads). HaCaT pBp-FGFR2c cultures show colony disorganization (**h**); the cortex is still evident (**i**, arrowheads), but peripheral cells display spindle shape and thin stress fibers (**i**, arrows). (**i**) Bar: 10μm; (**h**) Bar: 100μm. **(j)** Immunofluorescence using anti-Bek antibodies and TRITC-conjugated phalloidin was performed in HKs transiently tranfected with pCI-neo FGFR2b (HKs FGFR2b), pCI-neo FGFR2c (HKs FGFR2c) or pCI-neo empty vector (HKs pCI-neo) as control. HKs FGFR2c display a spindle-shaped morphology and actin stress fibers (Figure 1j, arrows), while HKs FGFR2b and control HKs pCI-neo maintain a cobblestone shape and peripheral actin cortex (arrowheads). Bar: 10μm.

In order to investigate the biological effects of the ectopic expression of the mesenchymal isoform of FGFR2 (FGFR2c) in human keratinocytes and to compare them to those obtained upon the overexpression of the epithelial isoform (FGFR2b), HaCaT cells were transiently transfected either with pCI-neo expression vector containing human FGFR2c (HaCaT FGFR2c) or human FGFR2b (HaCaT FGFR2b); cells transfected with the empty vector (HaCaT pCI-neo) and a primary culture of human fibroblasts (HFs), expressing endogenous levels of FGFR2b and FGFR2c respectively, were used as controls. Qualitative reverse transcriptase (RT)-PCR assay showed that specific FGFR2c amplicons were amplified only from complementary DNA (cDNA) of HaCaT FGFR2c transfected cells and, although less efficiently, from that of HFs, which express endogenous FGFR2c (Figure 1a). In contrast, the low levels of FGFR2b amplicons detected in HaCaT pCI-neo and in HaCaT FGFR2c cells, corresponding to endogenous FGFR2b, appeared strongly increased in HaCaT FGFR2b (Figure 1a). Amplification of 18S rRNA, used as internal control, was evident in all cells (Figure 1a). Transfection efficiency was then confirmed by quantitative real-time RT-PCR evaluating the relative levels of FGFR2 isoforms mRNA compared with 18S rRNA (Figure 1b). In agreement with the tissue specificity of the two splicing variants, FGFR2b mRNA appeared expressed at a lower level in all HaCaT cells and highly expressed in HaCaT FGFR2b cells; in contrast, FGFR2c mRNA was detected in HFs, highly expressed in HaCaT FGFR2c cells, but undetectable in HaCaT pCI-neo or HaCaT FGFR2b cells (Figure 1b). The enhanced expression of FGFR2 was validated at the protein level by western blot analysis using anti-Bek polyclonal antibodies C-17, which recognize the intracellular portion of the receptor and do not discriminate between the two splicing variants. The 140 KDa specific band corresponding to the receptor molecular weight was clearly visible upon the transfection of FGFR2 isoforms (Figure 1c). A very thin band was also visible in HFs and HaCaT pCI-neo cells corresponding to the endogenous expression of FGFR2c and FGFR2b isoforms respectively (Figure 1c). The alternative enhanced expression of FGFR2 isoforms at both mRNA and protein levels (Figure 1d and 1e respectively) was also verified in stable cultures (HaCaT pBp-FGFR2b, HaCaT pBp-FGFR2c) obtained transducing HaCaT cells as reported in materials and methods.

To verify the correct cellular localization of FGFR2b and FGFR2c isoforms, immunofluorescence examination was performed in transiently transfected cells. To this aim surface labeling at 4°C before fixation with anti-Bek polyclonal antibodies H-80, which recognize the extracellular portion of both receptor isoforms, or immunostaining of permeabilized cells with the above described anti-Bek polyclonal antibodies C-17 were carried out. Results showed that, similarly to FGFR2b, FGFR2c appeared mainly localized on the cell plasma membranes and in intracellular dots possibly corresponding to structures of the biosynthetic pathway (Figure 1f). Interestingly, cells highly expressing ectopic FGFR2c appeared elongated if compared to those overexpressing FGFR2b (Figure 1f), suggesting that the forced expression of the mesenchymal isoform of FGFR2 could impact on epithelial cell shape. In order to assess this possibility, a more detailed investigation was performed coupling immunofluorescence to low magnification phase contrast analysis. The results showed that while cells overexpressing FGFR2b appeared cobblestone-shaped and therefore morphologically indistinguishable to the surrounding ones (Figure 1g, arrowheads), cells ectopically expressing FGFR2c appeared spindle-shaped and detached from the neighboring ones (Figure 1g, arrows). Low magnification phase contrast analysis was performed also in HaCaT stable bulks and showed that the closely packed and compact colonies of polygonal cells observed in pre-confluent HaCaT pBp-FGFR2b cultures (Figure 1h) appeared more disorganized in HaCaT pBp-FGFR2c (Figure 1h), as a consequence of a greater tendency to isolated growth.

In order to further investigate the effects of FGFR2c expression on cell morphology, the organization of the actin cytoskeleton was analysed in stably transfected cells by fluorescence analysis using phalloidin-TRITC. The results showed that, in agreement with the compact appearance of their colonies, HaCaT pBp as well as HaCaT pBp-FGFR2b cultures displayed cobblestone-shaped and tightly packed cells in which the actin cytoskeleton appeared organized in peripheral cortical bundles (Figure 1i, arrowheads). In contrast, in HaCaT pBp-FGFR2c cultures, although the cortical bundles were still evident (Figure 1i, arrowheads), several peripheral cells appeared spindle-shaped, detached from the surrounding ones and with an actin cytoskeleton reorganized in thin stress fibers (Figure 1i, arrows). Thus, differently from FGFR2b overexpression, the ectopic expression of FGFR2c appears to affect HaCaT cell morphology and their ability to grow in compact colonies. To confirm these findings also in primary cultured cells, we analyzed in parallel experiments skin-derived normal human keratinocytes (HKs). Immunofluorescence performed as above showed that, while transiently transfected HKs FGFR2b and control HKs pCI-neo maintained a typical epithelial cell morphology with the actin cytoskeleton organized in a peripheral cortex (Figure 1j, arrowheads), cells ectopically expressing FGFR2c (HKs FGFR2c) displayed an elongated cell shape and actin cytoskeleton reorganization in stress fibers (Figure 1j, arrows).

**Figure 2:**
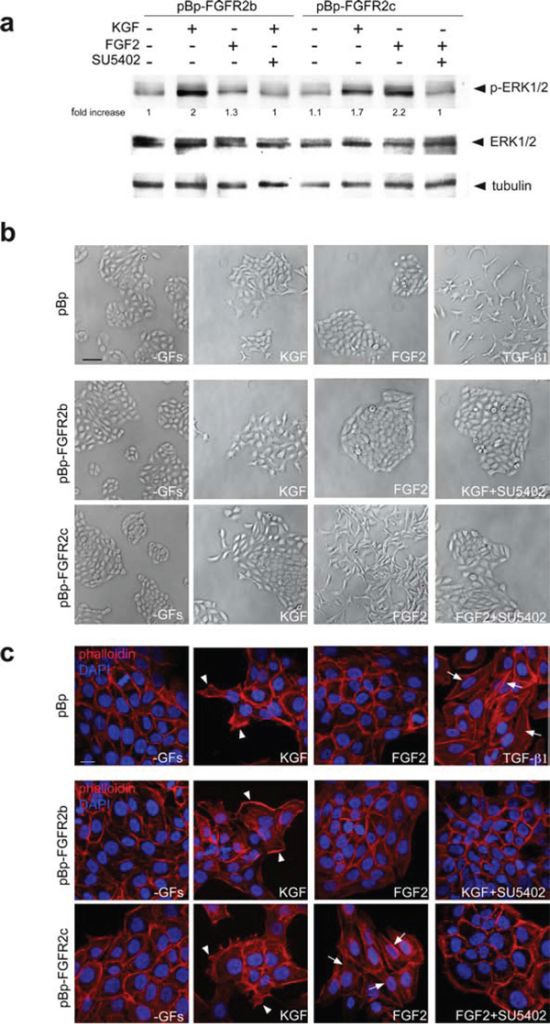
The expression of FGFR2c and its ligand-dependent activation trigger morphological and cytoskeletal changes reminiscent to EMT (**a**) HaCaT pBp-FGFR2b and HaCaT pBp-FGFR2c cells were serum starved and stimulated with KGF or FGF2 for 10 minutes in presence or not of the FGFR2 tyrosine kinase inhibitor SU5402. Western blot analysis shows that an evident phosphorylation of ERKs is induced in all cultures by KGF, while FGF2 triggers it only in HaCaT pBp-FGFR2c cells. ERK phosphorylation is decreased by treatment with SU5402. The equal loading was assessed using anti-a tubulin antibody. For densitometric analysis results are expressed as mean values from three independent experiments. (**b, c**) Phase contrast (**b**) and fluorescence analysis using phalloidin-TRITC (**c**) were performed in stably transduced cells stimulated with KGF or FGF2 for 24 h in presence or not of SU5402. Alternatively, HaCaT pBp cells were stimulated with TGF-b1 for 24 h. In all cultures the serum deprivation does not affect cell morphology or actin cytoskeleton organization, while KGF stimulation induces peripheral cell elongation and the appearance of lamellipodia and ruffles (c, arrowheads). Only in FGFR2c expressing cultures, FGF2 stimulation induces colony dispersion, spindle-shaped morphology and stress fiber formation (c, arrows). Similar phenotypic changes are observed in HaCaT pBp cells upon TGF-b1 stimulation (**b, c**, arrows). Both migratory and mesenchymal features are abrogated by SU5402. (**b**) Bar: 100μm; (**c**) Bar: 10μm.

Since it is well known that FGFRb and FGFRc isoforms respond to a different pattern of FGFs [1], we investigated whether the ectopically expressed FGFR2c can be functionally activated. To this aim, HaCaT pBp-FGFR2b and HaCaT pBp-FGFR2c cells were serum starved and alternatively stimulated with the FGFR2b specific ligand KGF/FGF7 or with FGF2, which does not bind to FGFR2b, but is able to activate other FGFRs, including FGFR2c. The triggering of the downstream signaling was evaluated analyzing the phosphorylation of the extracellular signal-related kinase 1 (ERK1) and the extracellular-signal related kinase 2 (ERK2) by western blot analysis. The results showed that, KGF induced ERK phosphorylation in both HaCaT pBp-FGFR2b and in HaCaT pBp-FGFR2c cells, which express endogenous FGFR2b (Figure 2a). In contrast, a strong ERK phosphorylation upon FGF2 stimulation was evident only in HaCaT pBp-FGFR2c cells (Figure 2a), strongly suggesting that it was a consequence of FGFR2c activation. The observation that either KGF-induced and the FGF2-dependent phosphorylation of ERKs were abolished by the specific FGFR2 tyrosine kinase inhibitor SU5402 (Figure 2a) confirmed this possibility.

In order to investigate the outcome of the ligand-dependent activation of FGFR2c on cell morphology, we analysed the effects induced by KGF or FGF2 stimulation. Phase contrast and fluorescence analysis using phalloidin-TRITC demonstrated that no appreciable changes were observed in all cultures upon serum deprivation; in fact, cells remained cobblestone-shaped, tightly packed (Figure 2b, c) and they conserved the actin cytoskeleton organized in peripheral cortical bundles (Figure 2c). Consistent with the widely demonstrated motogenic effect of KGF in keratinocytes [23–25] the stimulation with this growth factor induced the typical migratory phenotype in all cultures; cells located at the periphery of colonies displayed an elongated shape, (Figure 2b) lamellipodia and ruffles (Figure 2c, arrowheads) that were abrogated by SU5402 (Figure 2b, c). In contrast, FGF2 stimulation only impacted on HaCaT pBp-FGFR2c cell shape; the colonies appeared dispersed (Figure 2b), cells became clearly spindle-shaped and detached each others (Figure 2b), while the actin cytoskeleton appeared rearranged in tick stress fibers (Figure 2c, arrows) that disappeared in the presence of SU5402 (Figure 2c), as the consequence of FGFR2c shut-down. This mesenchymal phenotype was closely similar to that observed in HaCaT pBp cells upon the stimulation with TGF-b1 (Figure 2c, arrows), a well-established inductor of EMT in keratinocytes [18, 26]. Thus, FGFR2c expression forces HaCaT cells to detach each other and triggers cytoskeletal reorganization reminiscent to that occurring during EMT.

**Figure 3:**
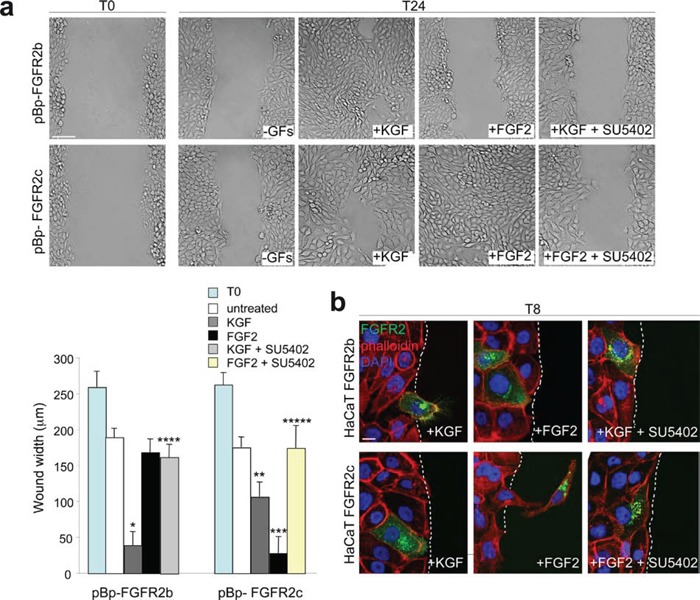
The ligand-dependent activation of FGFR2b and FGFR2c induces comparable migratory responses in keratinocytes (**a**) HaCaT pBp-FGFR2b and HaCaT pBp-FGFR2c cells were grown until confluence and a cell-free area was introduced in the monolayer using a tip. Cells were then immediately fixed (T0) or allowed to migrate for 24 h (T24) in the presence or not of KGF or FGF2. SU5402 was added to inhibit FGFR2 activity. The cell-free scratch area, evident at time 0 (T0), is partially repopulated by either untreated cells; the migratory response of pBp-FGFR2c cells to FGF2 is comparable to that observed in pBp-FGFR2b cells upon KGF stimulation. pBp-FGFR2c cells also shows a moderate response to KGF, while pBp-FGFR2b cells does not significantly respond to FGF2. Quantitative analysis of cell migration was assessed measuring the gap distance between the edges of the scratch area as reported in materials and methods. Results are expressed as mean value of three independent experiments ± standard error (SE). Student's t test was performed as reported in materials and methods and significance level has been defined as follows: *, **, *** p < 0.001 vs the corresponding untreated cells; **** p < 0.001 vs the corresponding KGF-treated cells; ***** p < 0.001 vs the corresponding FGF2-treated cells. Bar: 100μm. (**b**) Immunofluorescence analysis was performed using anti-Bek antibodies and phalloidin-TRITC in transiently transfected cells stimulated to migrate with KGF or FGF2 for 8 h in presence or not of SU5402. Migrating cells in response to KGF are visible in HaCaT FGFR2b cultures, while upon FGF2 stimulation fibroblast-like shaped cells invading the scratch area are detected only in HaCaT FGFR2c cultures. All ligand-induced features are abolished by SU5402. Bar: 10μm.

Previous studies by our group and others have demonstrated that KGF promotes keratinocyte migration [23–25], while it is well established that FGF2 induces migration of mesenchymal cells, including dermal fibroblasts [27]. In addition, the ectopic expression of FGFR2c makes epithelial cells able to migrate in response to FGF2 stimulation [11, 12]. Based on these evidences, we compared the ligand-dependent ability of FGFR2c versus FGFR2b to trigger keratinocyte motility using the “scratch assay” as previously described [23]. As expected [24], the stable overexpression of FGFR2b induced an increased cell migration in response to KGF (Figure 3a, upper panels) while no migratory effect was observed upon FGF2 stimulation (Figure 3a, upper panels). On the other hand, HaCaT pBp-FGFR2c cells displayed a migratory response to FGF2 which was comparable to that displayed by HaCaT pBp-FGFR2b cells in response to KGF (Figure 3a, bottom panels). Even if less evident, a motogenic response to KGF was also shown by cells expressing the FGFR2c, an effect that would be ascribed to the activation of endogenous FGFR2b by its specific ligand (Figure 3a, bottom panels). The treatment with SU5402 blocked all the ligand-dependent migratory responses (Figure 3a), confirming that FGFR2 isoform activation and signaling are required. Immunofluorescence analysis performed in transiently transfected cells using anti-Bek antibodies and TRITC-conjugated phalloidin, to visualize the F-actin cytoskeleton organization, showed that, consistently with the migration tendency as that observed during wound healing [23, 24], migrating cells in the scratch area were evident in HaCaT FGFR2b cultures upon short-time stimulation with KGF (Figure 3b, upper panels). In contrast, single cells invading the scratch area were observed in HaCaT FGFR2c cultures only upon FGF2 stimulation (Figure 3b, lower panels); when highly expressing FGFR2c, these cells appeared clearly detached from the neighbouring ones and they assumed a fibroblast-like shape (Figure 3b, lower panels) which is reminiscent of that observed in the single-cell migration during EMT [21, 22]. Again SU5402 abolished either KGF-mediated and FGF2-induced response as the consequence of FGFR2b and FGFR2c signalling shut-down (Figure 3b).

**Figure 4:**
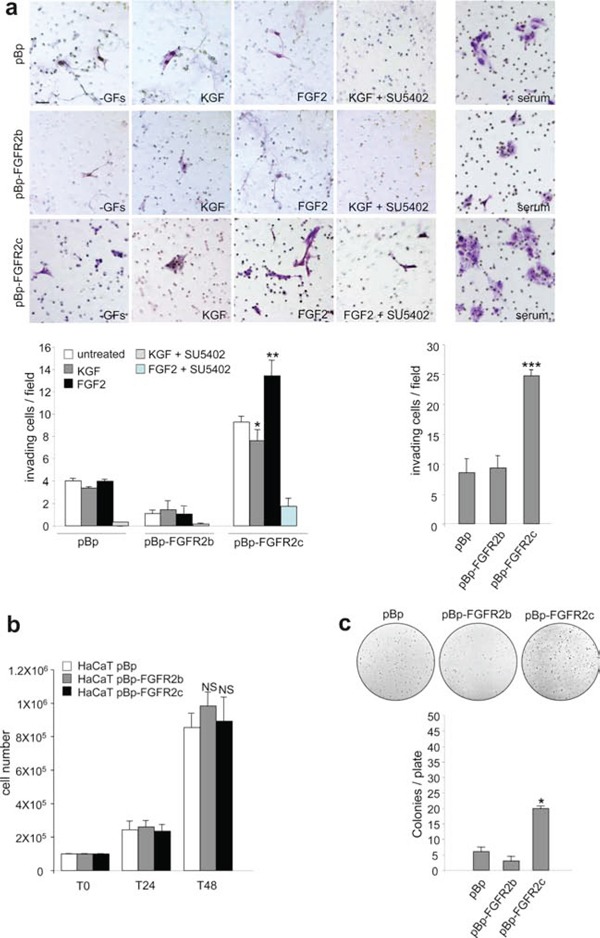
FGFR2c expression and signaling confer “in vitro” tumorigenic properties (**a**) HaCaT pBp, HaCaT pBp-FGFR2b and HaCaT pBp-FGFR2c cells were seeded on matrigel pre-coated transwell Boyden chamber filters. Cells were then serum starved and complete medium, KGF or FGF2 in presence or not of SU5402 were added in the bottom chamber to stimulate cell chemotaxis. In unstimulated conditions, FGFR2c expressing cultures appear more invasive compared to pBp or pBp-FGFR2b cells; moreover, FGF2 further enhances, while KGF represses, this tendency. The FGF2-induced invasive behavior is abolished by SU5402. Also in presence of complete medium, pBp-FGFR2c cultures appear significantly more invasive than pBp or pBp-FGFR2b cells. Quantitative analysis was assessed as reported in materials and methods. Results are expressed as mean values ± standard deviation (SD). Student's t test was performed as reported in materials and methods and significance level has been defined as follows: * p < 0.05 and ** p < 0.01 vs the corresponding untreated cells; *** p < 0.001 vs pBp cells. Bar: 20mm. (**b**) Proliferation assay performed in stably transduced cells grown in complete medium. At 24 h (T24) and 48 h (T48) from seeding, cells were harvested and counted as reported in materials and methods. No significant differences in cell proliferation were found at both 24 and 48 h time points. The results are expressed as the mean values of three independent counts ± SD. Student's t test was performed as reported in materials and methods and significance level has been defined as follows: NS vs HaCaT pBp cells. (**c**) Stably transduced cells were trypsinized and resuspended in medium containing agar as reported in materials and methods. Quantitative analysis performed after 2 weeks reveals that only pBp-FGFR2c cultures display a significant ability to form colonies in soft agar. Results are expressed as mean values ± SD. Student's t test was performed as reported in materials and methods and significance level has been defined as follows: * p < 0.05 vs pBp cells.

Thus, the expression and signalling of FGFR2b or FGFR2c induce HaCaT cell migration. However, our previous observations strongly indicated that only FGFR2c expression confers to these cells morphological features reminiscent to EMT. Based on this, we investigated the possible role of FGFR2c in triggering not only migration, but rather invasiveness ability in the well established non invasive HaCaT cells [28]. To this aim we analysed the capacity of HaCaT pBp-FGFR2c, pBp-HaCaT FGFR2b and HaCaT pBp cells to migrate through transwell Boyden chambers pre-coated with a thin layer of matrigel, a gel composed of reconstituted basement membrane elements resembling the basement membrane *in vivo*. Upon seeding, cells were serum starved and complete medium, KGF or FGF2 in presence or not of the FGFR2 inhibitor SU5402 were added in the bottom chamber for 48 hours in order to possibly stimulate cell chemotaxis. The results showed that the slightly increase of invading cells evident in unstimulated FGFR2c cultures compared to unstimulated pBp cultures (Figure 4a) was significantly increased by FGF2, but repressed by KGF stimulation (Figure 4a). In addition, in agreement with its tumor suppressive function in epithelial cells, FGFR2b expression further repressed the very poor tendency of HaCaT cells to migrate through the matrigel (Figure 4a). Also in cells stimulated with complete medium, FGFR2c cultures displayed a significant increase of invasive cells compared to control cells (Figure 4a). To assess whether cell proliferation could possibly contribute to the repopulation of the invaded side of the transwell membrane, the growth ability of the different stable transduced HaCaT cells in complete medium was assessed at the same time points in which the invasion experiments were carried out. The proliferation assay, performed on HaCaT pBp-FGFR2b and HaCaT pBp-FGFR2c as reported in materials and methods, showed no significant differences in cell number increase at 24 h or 48 h from seeding (Figure 4b).

The evidence that FGFR2c expression and signaling is able to confer invasive properties to HaCaT cells encouraged us to investigate also its possible role in inducing anchorage-independent growth, a crucial parameter to assess potential cell malignancy *in vitro*. The soft agar assay showed that, differently from cells overexpressing FGFR2b or control cells, FGFR2c cells displayed a significant ability to form colonies (Figure 4c).

Therefore, the morphological changes forced in HaCaT cells by FGFR2c expression are accompanied by the appearance of both invasiveness and anchorage-independent growth ability, all *in vitro* parameters that may suggest a potential tumorigenic role of FGFR2c in keratinocytes.

### FGFR2c expression and signaling induce modulation of EMT markers

**Figure 5:**
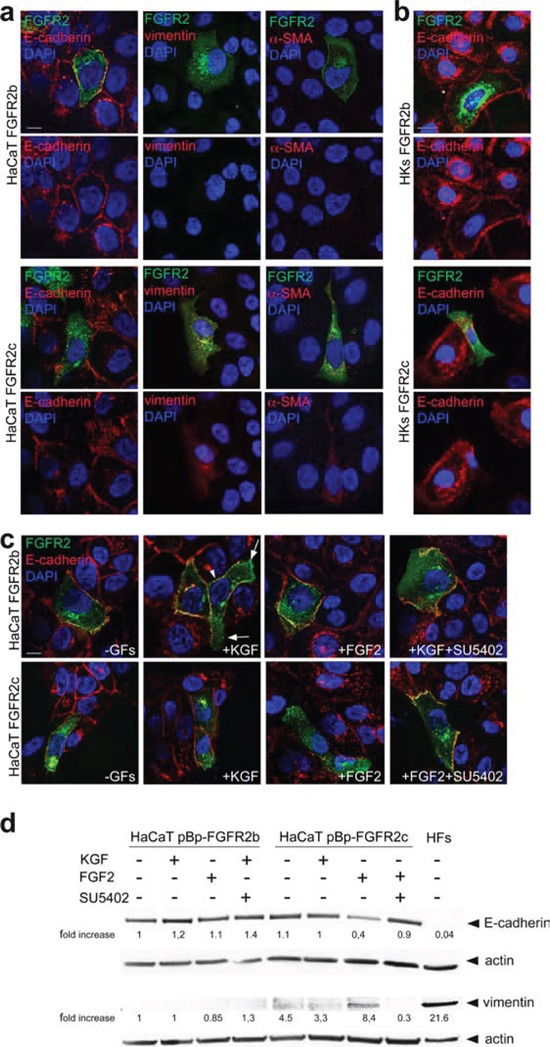
FGFR2c expression and signaling modulate epithelial and mesenchymal markers (**a, b**) HaCaT FGFR2b and HaCaT FGFR2c cells (**a**) as well as HKs FGFR2b and HKs FGFR2c (**b**) were grown in complete medium. Double immunofluorescence using anti-Bek polyclonal antibodies and anti-E-cadherin, anti-vimentin or anti-α-SMA monoclonal antibodies shows that, only in FGFR2c positive cells, the expression of E-cadherin is shut-down, while that of the two mesenchymal markers is slightly induced. Bar: 10 mm. (**c**) Transiently transfected cells were serum starved and stimulated with KGF or FGF2 for 24 h in presence or not of SU5402. Immunofluorescence performed as above reveals that the E-cadherin staining disappears in FGFR2c positive cells only upon FGF2 stimulation, but it is conserved when treatment is done in the presence of SU5402. E-cadherin signal is instead conserved on the plasma membranes of all FGFR2b positive cells; upon KGF stimulation the staining is visible in cell-cell contact sides (arrowhead), but it is lost at the leading edge (arrows). Bar: 10 mm. (**d**) Western blot analysis performed in stably transduced cells stimulated with growth factors as above, shows a reduction of E-cadherin band and the appearance of that corresponding to vimentin in pBp-FGFR2c cells only upon FGF2 stimulation. This opposite trend is abolished by SU5402. HFs were used as control. The equal loading was assessed using anti-b actin antibody. For densitometric analysis results are expressed as mean values from three independent experiments.

Based on the above observations that morphological changes induced by FGFR2c expression and signaling in keratinocytes are similar to those occurring during EMT, we wondered whether the ectopic expression of this mesenchymal isoform of FGFR2 would be able to alter the expression pattern of well recognized epithelial and mesenchymal biomarkers. Double immunofluorescence approaches using anti-Bek polyclonal and anti-E-cadherin monoclonal antibodies performed in transiently transfected HaCaT cells grown in complete medium demonstrated that the expression of epithelial marker E-cadherin was undetectable in cells ectopically expressing FGFR2c, while it was visible at the plasma membrane of the surrounding cells or in HaCaT FGFR2b (Figure 5a). Thus, the expression of E-cadherin appears unaffected by FGFR2b overexpression but it is strongly repressed by the ectopic expression of FGFR2c. Moreover, consistently with the hypothesis of a role for FGFR2c in the acquisition of a mesenchymal phenotype, immunofluorescence approach using anti-vimentin and anti-a smooth muscle actin (SMA) monoclonal antibodies showed that these mesenchymal markers were slightly induced in FGFR2c expressing cells, but remained undetectable in HaCaT FGFR2b (Figure 5a). In agreement with the observations obtained in HaCaT cells, also primary cultures of HKs expressing the FGFR2c mesenchymal isoform showed E-cadherin down-regulation (Figure 5b). In contrast, HKs overexpressing the epithelial FGFR2b isoform displayed E-cadherin staining on the plasma membrane similar to that observed in untransfected cells (Figure 5b). In order to determine whether the specific signaling of FGFR2c was required for E-cadherin repression observed in FGFR2c cells, treatment with KGF or FGF2 was also performed in presence or not of SU5402. The results showed that in HaCaT FGFR2b cells the plasma membrane expression of E-cadherin appeared unaffected by the treatments (Figure 5c): however, consistent with the conservation of this adherent-junction molecule during the collective cell migration in regeneration [22], after KGF stimulation, peripheral cells maintain E-cadherin staining on the plasma membrane in cell-cell contact sides (Figure 5b, arrowhead), but not at the leading edge side (Figure 5c, arrows), as previously described [29]. In contrast, this epithelial marker was undetectable in FGFR2c expressing cells after FGF2 stimulation (Figure 5c), while it remains visible in unstimulated cells or in cells stimulated with KGF (Figure 5c). The effect of FGF2 on E-cadherin down-modulation was abolished when the cells were stimulated in the presence of SU5402 (Figure 5b), indicating that the ligand-mediated activation of FGFR2c is required. On the other hand, upon treatment with FGF2 only FGFR2 expressing cells appeared positive for the mesenchymal marker α-SMA (Supplementary Figure 1).

The modulation of E-cadherin and vimentin expression was further investigated by western blot analysis. The results clearly showed a reduction of the epithelial marker and the appearance of the mesenchymal marker only in HaCaT cells stably expressing FGFR2c upon FGF2 stimulation (Figure 5d). Thus, the ectopic expression and ligand-mediated activation of FGFR2c in human keratinocytes induce morphological changes, F-actin cytoskeleton reorganization and epithelial/mesenchymal biomarker modulation. Since E-cadherin-based intercellular junctions are conserved during the collective migration occurring in wound healing, but are lost at the onset of EMT-related single-mesenchymal cell migration [22], these results strongly suggest that FGFR2c expression and signalling trigger EMT in keratinocytes.

**Figure 6:**
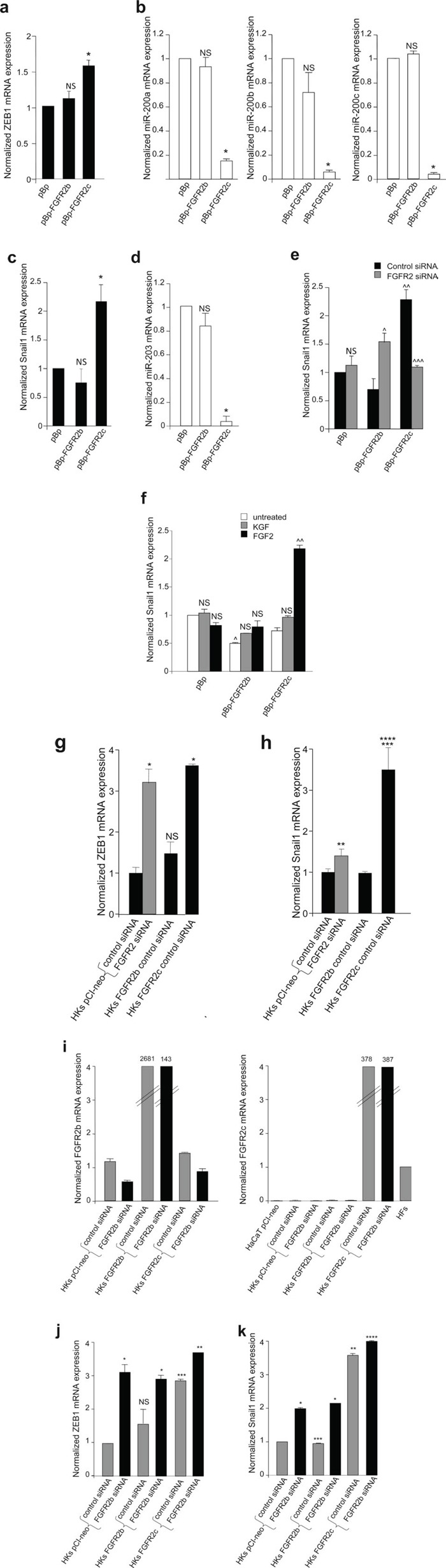
FGFR2c expression and signaling unbalance the transcription factors involved in EMT and their related miRs (**a**-**d**) Real-time RT-PCR analysis of the expression profile of ZEB1, Snail1 and related miRs performed in stably transduced cells grown in complete medium. The up-regulation of ZEB1 and Snail1 mRNAs correspond to a down-regulation of all miR-200 family members and miR-203 in FGFR2c expressing cells; no changes in the expression of transcription factor mRNAs or miRs are detected in FGFR2b overexpressing cells. (**e**) Stably transduced cells were transfected with small interfering RNA for FGFR2/Bek (FGFR2 siRNA), to obtain receptor silencing, or with an unrelated siRNA used as control. FGFR2 depletion abolishes Snail1 induction in pBp-FGFR2c cells, but enhances its expression in pBp-FGFR2b cells. (**f**) Stably transduced cells were serum starved and stimulated with growth factors as above; Snail1 mRNA expression is increased in FGFR2c expressing cells only after FGF2 stimulation. In unstimulated conditions, a significant reduction of Snail1 expression is evident in pBp-FGFR2b compared to control cells. (**g-k**) Real-time RT-PCR analysis of the expression profile of FGFR2b, FGFR2c, ZEB1 and Snail1 in HKs transiently transfected or co-transfected with the reported vectors and siRNAs. Primary cultured HFs and HaCaT pCI-neo were used as control. While FGFR2b overexpression does not appear to affect ZEB1 and Snail1 mRNA expression, their increase is evident either upon expression of FGFR2c or silencing of FGFR2 (**g h**) FGFR2b siRNA, which specifically down-regulates FGFR2b mRNA in cells endogenously-expressing or overexpressing the FGFR2b epithelial isoform (i) and does not interfere with FGFR2c expression (**i**) significantly increases both ZEB1 and Snail1 expression in HKs transfected with the empty vector as well as in those transfected with FGFR2b or FGFR2c (**j, k**). Results are expressed as mean value ± SE. Student's t test was performed as reported in materials and methods and significance level has been defined as follows: (**a**) NS and * p < 0.05 vs pBp cells; (**b**) NS and * p < 0.01 vs pBp cells; (**c, d**) NS and * p < 0.05 vs pBp cells; (**e**) ^ p < 0.01, ^^^ p < 0.05 and NS vs the corresponding control siRNA cells; ^^ p < 0.05 vs pBp cells; (**f**) NS and ^^ p < 0.05 vs the corresponding untreated cells; ^ p < 0.05 vs pBp cells. (**g, h**) NS, * p < 0.001, ** p < 0.005 and *** p < 0.05 vs HKs pCI-neo control siRNA; **** p < 0.05 vs HKs pCI-neo FGFR2 siRNA; (**j**) NS and *** p < 0.05 vs HKs pCI-neo control siRNA; * p < 0.01 and ** p < 0.05 vs the corresponding control siRNA HKs; (**k**) * p < 0.05 and **** NS vs the corresponding control siRNA HKs, ** p < 0.01 and *** NS vs HKs pCI-neo control siRNA

It is well known that changes in phenotypic features during EMT are the results of a complex gene reprogramming driven by different transcription factors [30, 31]. Given the role played by the transcription factor ZEB1 and by the negative feedback loop ZEB1/miR-200 in the repression of several epithelial markers, including E-cadherin, during EMT [30–32] the expression profile of this transcription factor and miR-200 family members was analysed in HaCaT stably expressing FGFR2b, FGFR2c or the empty vector pBp as control. Real-time RT-PCR showed that ZEB1 mRNA levels appeared slightly, but significantly, up-regulated by the ectopic expression of FGFR2c, while it remained unaffected by FGFR2b overexpression (Figure 6a). Consistent with the negative feedback loop existing between ZEB1 and miR-200, the levels of the three main miR-200 family members, miR-200a, miR-200b and miR-200c, appeared lower in FGFR2c expressing cells (Figure 6b).

Among the transcription factors which spatially and temporally cooperate during EMT [33], Snail1 is the widely recognized master for EMT transcription factor [30, 31], mainly associated with the initiation of the process [33]. Therefore, to assess whether FGFR2c expression is able to initiate EMT in keratinocytes, the expression of Snail1 was investigated in HaCaT cells expressing the two isoforms of FGFR2. The expression profile of miR-203 was also analyzed in parallel, since it has been recently demonstrated that Snail1/miR-203 double negative loop is involved in EMT [34]. Real-time RT-PCR performed in HaCaT cultures grown in complete medium and stably expressing FGFR2 isoforms or the empty vector demonstrated that the expression of FGFR2c is accompanied by a significant increase of Snail1 mRNA and a significant reduction of miR-203, while FGFR2b overexpression did not appear to affect them (Figure 6c, d). In order to demonstrate that the inductive effect on Snail1 observed in HaCaT cells stably expressing FGFR2c can be directly ascribed to FGFR2c expression, stable HaCaT pBp-FGFR2b and pBp-FGFR2c were transiently transfected with a specific FGFR2 small interfering RNA (FGFR2 siRNA), in order to silence FGFR2 gene and to obtain FGFR2 isoform depletion, or with an unrelated siRNA as control. The efficient depletion of the FGFR2 isoforms in FGFR2 siRNA-transfected cells was first verified at the protein level by western blot analysis using anti-Bek polyclonal antibodies (Supplementary Figure 2a). The real-time RT-PCR demonstrated that FGFR2 depletion blocked the increase of Snail1 mRNA observed in HaCaT pBp-FGFR2c cells (Figure 6e) indicating that the expression of FGFR2c is responsible for Snail1 induction. Interestingly, FGFR2 depletion appeared to induce a significant increase of Snail1 mRNA in HaCaT pBp-FGFR2b cells (Figure 6e), suggesting that, consistent with its tumor suppressive role, FGFR2b would exert a repressive role on this master inductor of EMT. Finally, the treatment with the specific growth factors showed that Snail1 mRNA was increased only following FGF2 stimulation of FGFR2c expressing cells (Figure 6f). Again, in agreement with its tumour suppressor role, the expression of FGFR2b isoform significantly repressed Snail1 transcript expression (Figure 6f). Thus, the ectopic expression of FGFR2c in human keratinocytes induces morphological and molecular modifications that are reminiscent of the EMT initiation and Snail1 appears to be the main transcription factor involved. Then, in order to verify whether the observed imbalance of either ZEB1 and Snail1 expression would occur also in primary cultures, the effects of FGFR2b and 2c overexpression were analysed in HKs. In addition, the impact of FGFR2 depletion was also analyzed. Interestingly, we found that the effects of the ectopic expression of FGFR2c were much more evident (up to three fold increase for both ZEB1 and Snail1) if compared to the effects observed in HaCaT cells (Figure 6g, h). Moreover, differently from that found in the cell line (see Figure 6e), the generic silencing of FGFR2 was able to increase both ZEB1 and Snail1 expression already in untransfected cells (Figure 6g, h), further supporting the hypothesis of the tumor suppressive role of the endogenously expressed FGFR2b. In addition, the impact of FGFR2c expression in FGFR2b-depleted HKs was also investigated through specific FGFR2b silencing. Real-time RT-PCR analysis confirmed that FGFR2b siRNA was able to down-regulate FGFR2b mRNA expression without interfering with that of FGFR2c (Figure 6i) and the efficient FGFR2b depletion in siRNA-transfected cells was verified also at the protein level by western blot analysis using anti-Bek polyclonal antibodies (Supplementary Figure 2b). We found that FGFR2b silencing significantly enhanced both ZEB1 and Snail1 mRNA expression either in HKs expressing endogenous FGFR2b or in cells overexpressing both FGFR2b and FGFR2c (Figure 6j, k). Interestingly, in cells ectopically expressing FGFR2c, the effect induced by FGFR2b siRNA on Snail1 expression (Figure 6k) appeared opposite to that exerted by the general FGFR2 siRNA (see Figure 6e) targeting both FGFR2b and FGFR2c. These results strongly indicate that FGFR2b and FGFR2c are able to play opposite roles in the epithelial and mesenchymal cell behaviour.

**Figure 7:**
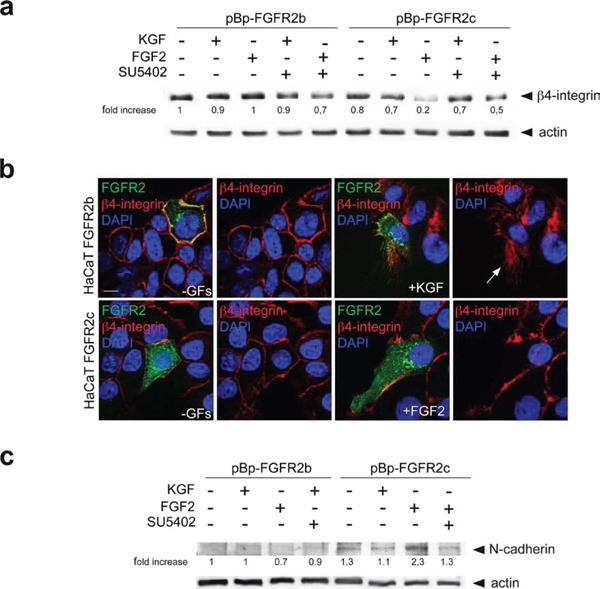
FGFR2c expression and signaling impact on β4-integrin and N-cadherin expression pattern HaCaT cells stably and transiently expressing the FGFR2 isoforms were stimulated with KGF and FGF2 in presence or not of SU5402 as reported above. (**a**) Western blot analysis shows that the band corresponding to *β*4-integrin is strongly attenuated only in HaCaT pBp-FGFR2c cells upon FGF2 stimulation, but it is conserved in the presence of SU5402. The equal loading was assessed using anti-b actin antibody. For densitometric analysis results are expressed as mean values from three independent experiments. (**b**) Double immunofluorescence analysis performed in transiently transfected cells shows that b4-integrin is uniformly distributed on the plasma membranes of all unstimulated cells, while its staining translocates at the leading edge of migrating FGFR2b cells upon KGF stimulation and it is lost in FGFR2c positive cells upon FGF2 stimulation. (**c**) Western blot analysis performed in cells stably expressing FGFR2 isoforms clearly shows that N-cadherin band appears only in FGF2-stimulated pBp-FGFR2c cells, but remains undetectable upon FGF2 stimulation in the presence of SU5402. The equal loading was assessed using anti-b actin antibody. For densitometric analysis results are expressed as mean values from three independent experiments.

In order to confirm the nature of the biological features triggered in human keratinocytes by the ectopic expression of FGFR2c and to better distinguish these features from those occurring during wound healing, we analyzed the expression pattern of discriminating markers. Since the previously considered EMT transcription factor Snail1 does not appear modulated in keratinocytes during wound healing [29, 35], our above observation of Snail1 up-regulation in cells expressing FGFR2c already indicated that the morphological changes and gene reprogramming observed in these cells correspond to EMT. To further validate this possibility, our attention was focused on the expression profiles of other epithelial/mesenchymal biomarkers discriminating between wound healing and EMT [36]. Western blot analysis on stably expressing cells demonstrated that the protein level of the hemidesmosome component b4-integrin, an epithelial marker that persists in migrating keratinocytes [37–40] but disappears during EMT [40], was strongly reduced only in HaCaT pBp-FGFR2c upon FGF2 stimulation (Figure 7a). Double immunofluorescence approaches using anti-Bek polyclonal antibodies and anti-b4-integrin monoclonal antibody showed that the plasma membrane staining corresponding to the hemidesmosome component was evident in all unstimulated cultures (Figure 7b) as well as in HaCaT FGFR2b cells upon KGF stimulation, where it appeared translocated at the leading edge of migrating cells (Figure 7b, arrow), as expected [37], [39]. Consistent with our hypothesis, b4-integrin staining disappeared in FGFR2c cells upon FGF2 stimulation, suggesting that the ectopic expression and signaling of FGFR2c are responsible for b4-integrin shut-down (Figure 7b). In parallel we also analyzed the expression of the cell-cell junction component N-cadherin, which is induced in EMT, but remains unexpressed during epithelial cell motility [36]: western blot analysis clearly showed that this mesenchymal marker displayed an opposite trend compared to that observed for b4-integrin, since it appeared expressed only in FGF2-treated HaCaT pBp-FGFR2c cells (Figure 7c) and remained quite undetectable in all other conditions (Figure 7c).

Taken together our results suggest that the ectopic expression and activation of FGFR2c in HaCaT cells triggers a process corresponding to initiation of the pathological type III EMT, but not to the more physiological type II EMT occurring during wound healing, which is in turn induced by activation of the epithelial isoform FGFR2b.

## DISCUSSION

Aim of this study was to analyze the phenotypic and behavioural impact of the aberrant expression of FGFR2c in normal epithelial cells carrying also endogenous FGFR2b. Our morphological investigations demonstrated that, differently from FGFR2b overexpression, the ectopic expression of FGFR2c and its ligand-dependent activation force non-tumorigenic keratinocytes to acquire a mesenchymal-like shape and to reorganize the actin cytoskeleton in thick stress fibers. These morphological changes result in colony disorganization, cell-cell detachment and tendency to isolated growth, all features which are strongly reminiscent to those occurring during EMT. Consistent with this possibility, we also found that the observed phenotypic switch was accompanied by the down-regulation of epithelial and appearance of mesenchymal biomarkers. In addition, the regulatory loops between specific transcription factors, such as ZEB1 and Snail1, and their related miRs were clearly unbalanced toward EMT. Since in our *in vitro* model FGFR2c expressing cells retain the expression of the endogenous FGFR2b, we may suggest that FGFR2c is able to activate in normal keratinocytes unknown molecular mechanisms that force them to undergo EMT overcoming the tumor suppressive activity of the endogenous FGFR2b. The molecular mechanisms underlying the different biological outcome of the expression and ligand-specific activation of FGFR2b and 2c isoforms remain to be defined. Among the possible signaling pathways, which would be more affected by the aberrant expression of a mesenchymal receptor in an epithelial cell context, the transient vs sustained activations of ERK1/2 or Akt could be good candidates, since they appear to be involved in the FGFR-mediated control of proliferation, survival, differentiation and migration [41] and they represent the target for new anti-cancer therapies [42].

EMT is a single definition, which actually includes different physiological and pathological manifestations. For this reason we also carried out the analysis of discriminating markers, which strongly indicated, for the FGFR2c-driven EMT, a specific expression signature corresponding to the pathological type III EMT, but not to the physiological type II EMT, which is instead induced by FGFR2b activation during wound healing. Being type III EMT associated to tumor progression [19, 20], our finding that FGFR2c-expressing keratinocytes show either anchorage-independent growth ability and invasiveness appear to strongly suggest that FGFR2c might contribute to the early steps of carcinogenesis. This possibility had already been advanced by us in a recent study performed using the early protein E5 of human papilloma virus 16 (16E5) as a powerful tool for perturbing the epithelial homeostasis [9, 43]. In the wake of evidences, indicating that the switch from epithelial to mesenchymal isoforms of FGFRs is associated with induced EMT [17], we demonstrated that 16E5 specifically unbalances the FGFR2 splicing machinery toward FGFR2c expression and induces EMT in normal keratinocytes [18]. Being 16E5 a very early expressed protein during HPV16 infection, this work encouraged us to propose that, at least in cervical cancer, the altered FGFR2 splicing and the consequent appearance of FGFR2c might play an important role in early tumor development. Actually, this assumption appears also true for the renal carcinoma context in which it has been recently demonstrated that the aberrant expression of FGFR2c not only correlates with a mesenchymal/EMT signature, but also with enhanced tumor growth and invasion [13]. Consistently, recent findings obtained in different carcinoma cell lines have shown that the induced expression of FGFR2c in tumor cells poorly expressing this FGFR2 variant correlates with enhanced *in vitro* tumorigenicity and invasiveness, while its depletion in highly-expressing cells represses these features [10–12]. Moreover, Toriseva et al. [44] found that the expression of FGFR2b is attenuated in human epidermal SCCs compared to healthy skin and that its ligand-dependent activation suppresses the malignant phenotype as well as tumor progression. Despite these reports, the role of each FGFR2 isoform in carcinomas remains controversial: FGFR2b has been identified as the FGFR2 isoform possibly involved in SCC anchorage-independent growth [45] and FGFR2c may act as a possible driver of mesenchymal-epithelial transition (MET) during metastatic cascade [15]. Our future work will be focused on this crucial topic through the analysis of the functional effects of specific silencing of FGFR2b and 2c isoforms in selected human SCC cell lines showing opposite signatures of FGFR2b and FGFR2c expression.

The hypothesis of FGFR2c involvement in EMT and consequently in early tumor development is also strengthened by the interesting findings of Oltean and coworkers [16] which, using an *in vivo* model of epithelial tumor progression, demonstrated that the inactivation of the FGFR2 splicing machinery, and consequently the aberrant appearance of FGFR2c, is associated to mesenchymal/EMT phenotype in primary tumor cells, while its restoration, which drives to FGFR2c down-regulation in favor of FGFR2b reappearance, is correlated with the epithelial/MET phenotype observed in micrometastases. These authors proposed the exciting possibility that the phenotypic versatility of malignant tumor cells might directly derive from FGFR2 splicing decisions, which enable them to promptly respond to different microenvironmental signals. In agreement with these results, we observed that a pronounced EMT and invasive phenotype are evident in FGFR2c-expressing keratinocytes only upon FGF2 stimulation, suggesting that the ligand-dependent activation of this receptor variant is required. Therefore, our observations strongly reinforce the assumption that the aberrant expression of FGFR2c could represent a crucial upstream event able to alter the response of normal cells to stromal growth factors, unbalancing the receptor signaling. In conclusion, we may speculate that, independently from the retention of low levels of FGFR2b expression, the appearance of FGFR2c impacts on the potential phenotypic plasticity of non-transformed epithelial cells, driving the early steps of their transition toward mesenchymal and tumorigenic features.

## MATERIALS AND METHODS

### Cells and treatments

The human keratinocyte cell line HaCaT [28] and the human embryonic kidney cell line Phoenix Amphotrophic (AMPHO) (ATCC) were cultured in Dulbecco's modified Eagle's medium (DMEM), supplemented with 10% fetal bovine serum (FBS) plus antibiotics. Primary cultures of human keratinocytes and human fibroblasts derived from healthy skin (HKs and HFs respectively) were obtained from patients attending the Dermatology Unit of the Sant'Andrea Hospital of Rome; all patients were extensively informed and their consent for the investigation was given and collected in written form in accordance with guidelines approved by the management of the Sant'Andrea Hospital. Primary cells were isolated and cultured as previously described. [46, 47].

HaCaT cells were transiently transfected with pCI-neo empty vector (HaCaT pCI-neo), with pCI-neo containing human FGFR2b/KGFR (HaCaT FGFR2b) [48, 49] or with human FGFR2c (HaCaT FGFR2c) using jetPEI^TM^ DNA Transfection Reagent (Polyplus-transfection, New York, NY) according to the manufacturer's instructions. The cDNA coding for FGFR2c was amplified by pMD18T-simple FGFR2c (HG10824-M, Sino Biological Inc., Beijing, China) and subcloned in pCI-neo expression vector using standard procedures (Promega, Madison, WI) and the resulting pCI-neo FGFR2c construct was verified by sequencing.

To generate HaCaT cells stably expressing FGFR2c or overexpressing FGFR2b, HEK293T Phoenix packaging cells were transfected with pBABE-Puro (pBp) empty vector (Addgene, Cambridge, MA, plasmid #51070), with pBp-FGFR2b-WT (Addgene, plasmid #45698) [50] or with pBp-FGFR2c-WT (Addgene, plasmid #45699) [50] retroviral expression vectors using jetPEI^TM^ DNA Transfection Reagent (Polyplus-transfection) as above. After 12 h from transfection, the medium was replaced with fresh growth medium and supernatants were collected after additional 24 h and 48 h. HaCaT cells were then transduced with the different supernatants in presence of polybrene 4 μg/ml (Sigma-Aldrich Inc., Saint Louis, MO) and selection for HaCaT pBp, HaCaT pBp-FGFR2b or HaCaT pBp-FGFR2c stable bulks was carried out using puromycin 1 μg/ml (Sigma).

For RNA interference and consequent *FGFR2* silencing, cells were transfected with Bek small interfering RNA (FGFR2 siRNA) (Santa Cruz Biotechnology, Santa Cruz, CA) or with a specific FGFR2b siRNA sequence (5′-AATTATATAGGGCAGGCCAAC-3′) (Qiagen, Valencia, CA) [51] using Lipofectamine 2000 Transfection Reagent (Invitrogen, Carlsbad, CA) according to the manufacturer's protocol. Transfection with a specific FGFR2c siRNA sequence (5′-GGAATGTAACTTTTGAGGA-3′) [52] or with a control sequence (5′-AATTCTCCGAACGTGTCACGT-3′) (Qiagen), were also performed.

For growth factors stimulation, cells were serum starved or incubated with FGF7 (Upstate Biotechnology, Lake Placid, NY) or with FGF2 (PeproTech, London, UK) 25 ng/ml for 24 h or 48 hr at 37°C. Alternatively, cells were treated with TGF-β1 (PeproTech) 10 ng/ml for 24 h at 37°C. To induce activation and signaling of FGFR2 isoforms, cells were serum starved and incubated with FGF7 (Upstate Biotechnology) or FGF2 (PeproTech) 100 ng/ml for 10 min at 37°C. For inhibition of FGFR2b and FGFR2c tyrosine kinase activity, cells were pre-incubated with a specific FGFR2 tyrosine kinase inhibitor, SU5402 25 μM (Calbiochem, Nottingham, UK) for 1 h before treatments with growth factors (GFs).

### Proliferation assay

HaCaT pBp, HaCaT pBp-FGFR2b and HaCaT pBp-FGFR2c were seeded in 6-cm culture plates at a density of 1 ×10^5^ cells per plate (T0) and left to grow in complete medium. After 24 h (T24) and 48 h (T48), cultures were harvested by incubation in 0.05% trypsin, 0.02% ethylenediamine tetraacetic acid (EDTA) for 15 min at 37°C, and the cell counts were performed using a hemocytometer. The results are expressed as the mean values of three independent counts ± standard deviation (SD). Student's t test was performed and significance levels have been defined as p < 0.05.

### Scratch assay

HaCaT cells transiently and stably expressing FGFR2b or FGFR2c were seeded at 2×10^5^ cells on 35 mm plates and grown until confluence. Confluent cells were serum starved for 12 hr and then a standardized cell-free area was introduced by scraping the monolayer with a sterile tip, as previously described [23]. Some plates were fixed and photographed immediately after scratching, representing a T0 control. After intensive wash, cells were incubated for 12 h with FGF7 or FGF2 in the presence or absence of SU5402. Cells were fixed with 4% paraformaldehyde and processed for immunofluorescence. Both phase contrast and immunofluorescence images were taken using an ApoTome System (Zeiss) connected with an Axiovert 200 inverted microscope (Zeiss). Cell migration was quantitated by measuring the gap distance between the scratch edges (three different measures for each photograph), using the Axiovision software (Zeiss). The results are expressed as the mean values of three independent experiments ± standard error (SE). Student's t test was performed and significance levels have been defined as p < 0.05.

### Invasion assay

Migration assay was performed using 24-well transwell migration Boyden chambers (8 μm pore size; Costar, Cambridge, MA, USA) precoated with matrigel (dilution 1:2 in DMEM; BD Biosciences, Bedford, MA, USA). 5×10^4^ cells were seeded in each filter and serum starved for 4 h at 37°C. To induce chemotaxis: complete medium, KGF 25 ng/ml or FGF2 25 ng/ml in presence or not of SU5402 as above were added to the lower chamber. After 48 h, cells on the upper side of membranes were removed, while cells migrated on the bottom side were fixed in methanol and stained with toluidine blue. Quantitative analysis was assessed counting for each sample the migrated cells in 10 microscopic fields (magnification: 200-fold) from three independent experiments. Results have been expressed as mean values ± SD. p values were calculated using Student's t test and significance level has been defined as p > 0.05.

### Soft agar colony formation assay

Cells were trypsinized and resuspended (1.5×10^4^ cells) in 3 ml of complete medium containing 0.4% agar. Cell suspension was added to 0.6% agar layer in six-well plates. Immediately after plating, only single cells were visible in each well. After two weeks, quantitative analysis was performed counting for each sample only >50 cells soft agar colonies from three independent experiments. Results have been expressed as mean values ± SD. p values were calculated using Student's t test and significance level has been defined as p > 0.05.

### Immunofluorescence

HaCaT cells, grown on coverslips, were fixed with 4% paraformaldehyde in PBS for 30 min at 25°C followed by treatment with 0.1 M glycine for 20 min at 25°C and with 0.1% Triton X-100 for additional 5 min at 25°C to allow permeabilization. Cells were then incubated for 1 h at 25°C with the following primary antibodies: rabbit polyclonal anti-Bek (1:50 in PBS; C-17, Santa Cruz) directed against the intracellular portion of FGFR2 isoforms, rabbit polyclonal anti-Bek (1:50 in PBS; H-80, Santa Cruz) directed against the extracellular portion of FGFR2 isoforms and mouse monoclonal anti-E cadherin (1:50 in PBS; NCH-38, Dako, Carpinteria, CA), mouse monoclonal anti-α-SMA (1:100 in PBS; 1A4, Dako), mouse monoclonal anti-vimentin (1:50 in PBS; V9, Dako), mouse monoclonal anti-β4 integrin (1:50 in PBS; Santa Cruz). The primary antibodies were visualized using goat anti-mouse IgG-Texas Red (1:200 in PBS; Jackson Immunoresearch Laboratories, West Grove, PA), goat anti-rabbit IgG-Texas Red (1:200 in PBS; Jackson Immunoresearch Laboratories), goat anti-rabbit IgG-FITC (1:400 in PBS; Cappel Research Products, Durham, NC) for 30 min at 25°C. Actin cytoskeleton was visualized using TRITC-phalloidin (1:100 in PBS; Sigma). Nuclei were stained with DAPI (1:1000 in PBS; Sigma). Coverslips were finally mounted with mowiol (Sigma) for observation.

Fluorescence signals were analyzed by scanning cells in a series of sequential sections with an ApoTome System (Zeiss, Oberkochen, Germany); image analysis was performed by the Axiovision software (Zeiss) and 3D reconstruction of a selection of three central optical sections was shown in each figure.

### Western blot analysis

Cells were lysed in a buffer containing 50 mM HEPES, pH 7.5, 150 mM NaCl, 1% glycerol, 1% Triton X-100, 1.5 mM MgCl_2_, 5 mM EGTA, supplemented with protease inhibitors (10 μg/ml aprotinin, 1 mM PMSF, 10 μg/ml leupeptin), and phosphatase inhibitors (1 mM sodium orthovanadate, 20 mM sodium pyrophosphate, 0.5 M NaF). A range between 20 and 50 μg of total protein was resolved under reducing conditions by 8 or 12% SDS-PAGE and transferred to reinforced nitrocellulose (BA-S 83, Schleider and Schuell, Keene, NH). The membranes were blocked with 5% nonfat dry milk in PBS 0.1% Tween 20 and incubated with anti-Bek polyclonal antibodies (C-17, Santa Cruz), anti-N-cadherin polyclonal antibodies (Novus Biologicals, Littleton, CO), anti-p44/42 MAPK (ERK1/2) monoclonal antibody (137F5, Cell Signaling technology, Beverly, MA), anti-P-p44/42 MAPK (P-ERK1/2) monoclonal antibody (E10, Cell Signaling), anti-E-cadherin monoclonal antibody (NCH-38, Dako), anti-vimentin monoclonal antibody (V9, Dako), anti-β4 integrin monoclonal antibody (7, Santa Cruz), followed by enhanced chemiluminescence detection (ECL, Amersham, Alington Heights, IL). The membranes were rehydrated and probed again with anti-β-actin (AC-15, Sigma) monoclonal antibody or anti-α-Tubulin (B7, Santa Cruz) monoclonal antibody to estimate the protein equal loading. Densitometric analysis was performed using Quantity One Program (Bio-Rad Laboratories, Hercules, CA, USA). Results from three different experiments were normalized, expressed as fold increase respect to the control value and reported as mean values.

### Primer

Oligonucleotide primers for target genes and for the housekeeping gene were chosen with the assistance of the Oligo 5.0 computer program (National Biosciences, Plymouth, MN) and purchased from Invitrogen. The following primers were used: for *FGFR2b/KGFR* target gene: 5′-CGTGGAAAAGAACGGCAGTAAATA-3′ (sense), 5′-GAACTATTTATCCCCGAGTGCTTG-3′ (anti-sense); for *FGFR2c* target gene: 5′-TGAGGACGCTGGGGAATATACG-3′ (sense), 5′-TAGTCTGGGGAAGCTGTAATCTCCT-3′ (anti-sense); for *Snail1* target gene: 5′-GCTGCAGGACTCTAATCCAGA-3′ (sense), 5′-ATCTCCGGAGGTGGGATG-3′ (antisense); for *ZEB1* target gene: 5′-GGGAGGAGCAGTGAAAGAGA-3′ (sense), 5′-TTTCTTGCCCTTCCTTTCTG-3′ (antisense); for the 18S rRNA housekeeping gene 5′-AACCAACCCGGTCAGCCCCT-3′ (sense), 5′-TTCGAATGGGTCGTCGCCGC-3′ (antisense). For each primer pair, we performed no-template control and no-reverse-transcriptase control (RT negative) assays, which produced negligible signals. For microRNA Taqman assays, primers and probes were provided by Applied Biosystems (Applied Biosystems, Foster City, CA).

### RNA extraction and cDNA synthesis

RNA was extracted using the TRIzol method (Invitrogen) according to manufacturer's instructions and eluted with 0.1% diethylpyrocarbonate (DEPC)-treated water. Each sample was treated with DNAase I (Invitrogen). Total RNA concentration was quantitated by spectrophotometry; 1 μg of total RNA was used to reverse transcription using iScript^TM^ cDNA synthesis kit (Bio-Rad, Hercules, CA) according to manufacturer's instructions. For microRNA Taqman assays, 2.5 ng of total RNA were reverse transcribed using Taqman® MicroRNA Reverse Transcription Kit (Applied Biosystems).

### PCR amplification and real-time quantitation

Real-Time PCR was performed using the iCycler Real-Time Detection System (iQ5 Bio-Rad) with optimized PCR conditions. The reaction was carried out in 96-well plate using iQ SYBR Green Supermix (Bio-Rad) adding forward and reverse primers for each gene and 1 μl of diluted template cDNA to a final reaction volume of 15 μl. All assays included a negative control and were replicated three times. The thermal cycling program was performed as described [8]. The PCR products were analyzed on 2.0% agarose gel, stained with ethidium bromide and visualized under ultraviolet light. Real-time quantitation was performed with the help of the iCycler IQ optical system software version 3.0a (Bio-Rad), according to the manufacturer's manual.

Relative quantities of mature microRNAs were determined using Applied Biosystems TaqMan microRNA Assays (Applied Biosystems). Results are reported as mean ± SE from three different experiments in triplicate. Student's t test was performed and significance levels have been defined as p < 0.05.

## SUPPLEMENTARY FIGURES


