# Supplementary material for: Expression of the FGFR2 mesenchymal splicing variant in epithelial cells drives epithelial-mesenchymal transition
Source: Oncotarget. 2015 Dec 21;7(5):5440–60. doi: 10.18632/oncotarget.6706 (PMC4868697; doi:10.18632/oncotarget.6706)
Supplement: Supplementary file 1 [file oncotarget-07-5440-s001.pdf]

SUPPLEMENTARY FIGURES

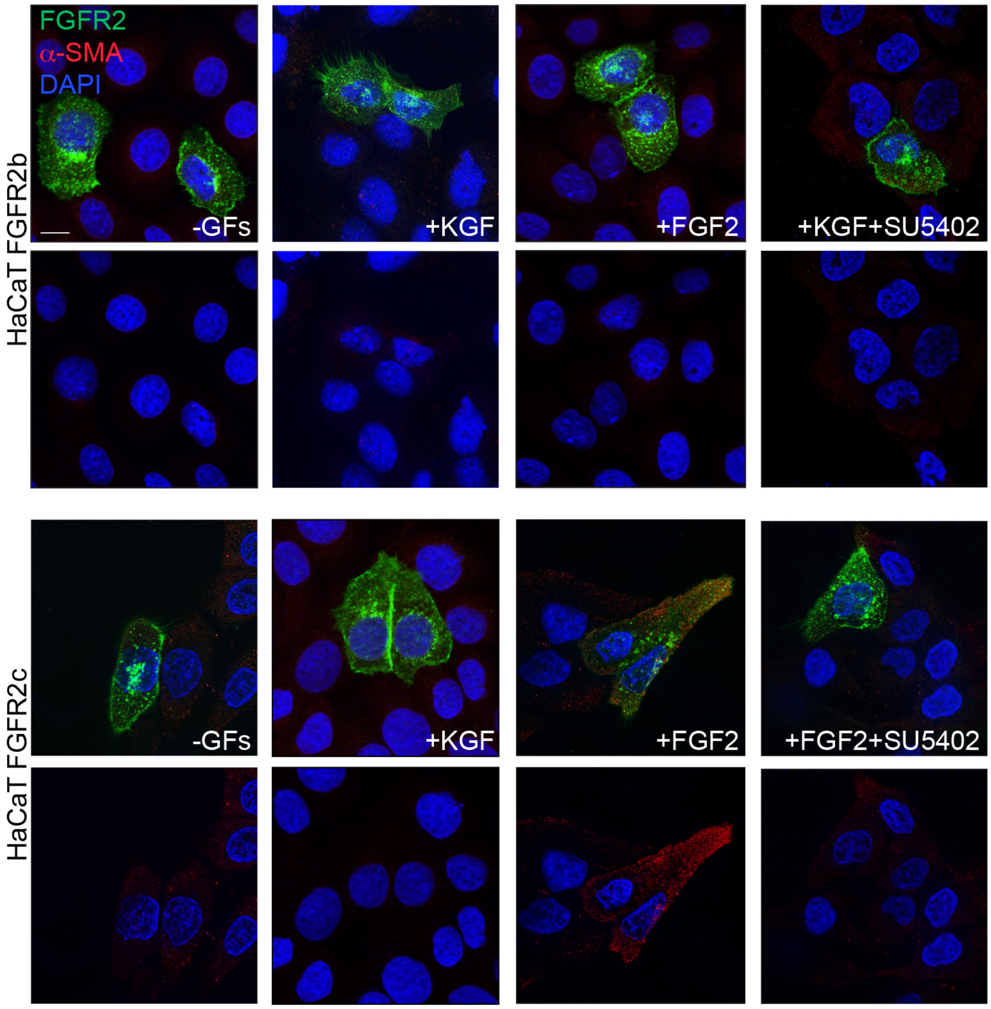

Supplementary Figure S1: FGFR2c expression and signaling induces the appearance of the mesenchymal marker α-SMA. Transiently transfected cells were serum starved and stimulated with KGF or FGF2 in presence or not of SU5402. Double immunofluorescence using anti-Bek polyclonal antibodies and anti-α-SMA monoclonal antibody shows that the appearance of α-SMA staining in FGFR2c cells is visible only upon FGF2 stimulation, but it remains undetectable when the treatment is performed in the presence of SU5402. Bar: 10 μm.

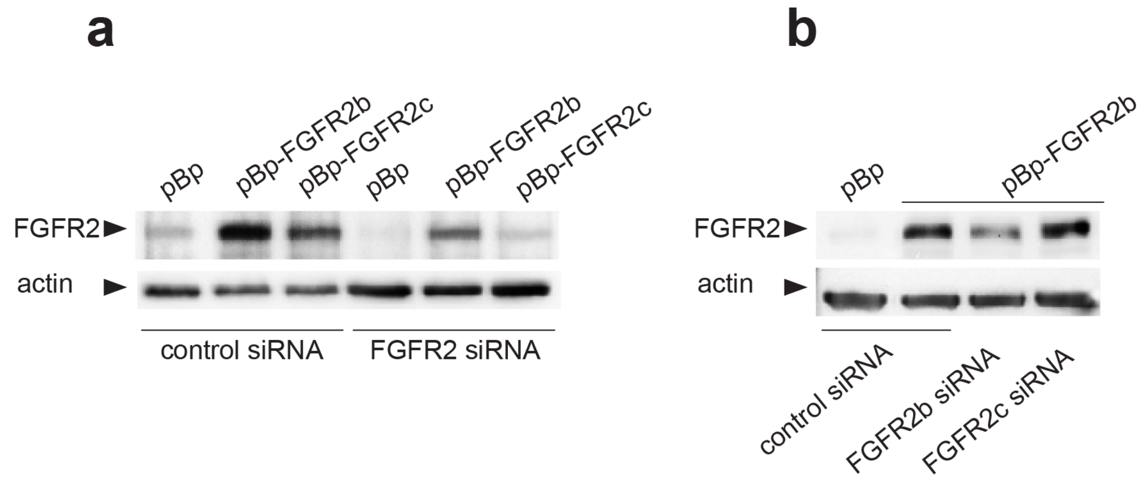

Supplementary Figure S2: Transfection with FGFR2 siRNA and with FGFR2b si RNA down-regulates FGFR2 and FGFR2b protein expression, respectively. HaCaT pBp, HaCaT pBp-FGFR 2b and HaCaT pBp-FGFR2c cells were transfected with small interfering RNA for FGFR2/Bek (FGFR2 siRNA) (**a**), to obtain the silencing of all receptor isoforms, or with a specific siRNA for FGFR2b isoform (FGFR2b si RNA) (**b**), to obtain the receptor epithelial isoform silencing. Unrelated siRNAs and a specific siRNA for FGFR2c isoform (FGFR2c si RNA) were used as control. Western blot analysis using anti-Bek antibodies reveals that FGFR2 siRNA induces an efficient depletion of FGFR2 in all samples. FGFR2b si RNA, but not FGFR2c si RNA down.regulates FGFR2b expression in HaCaT pBp-FGFR2b cells. The equal loading was assessed using anti-β actin antibody.
